# Adult acute respiratory distress syndrome due to human parvovirus B19 infection after cardiac surgery: a case report

**DOI:** 10.1186/s12879-022-07213-9

**Published:** 2022-03-07

**Authors:** Min Ma, Xiaojun Ma, Ming Jia, Xiaotong Hou, Hong Wang

**Affiliations:** 1 Center for Cardiac Intensive Care, Beijing Anzhen Hospital, Capital Medical University, Beijing Institute of Heart Lung and Blood Vessel Diseases, 2 Anzhen Road, Chaoyang District, Beijing, 100029 China; 2 Department of Infectious Diseases, Peking Union Medical College Hospital, Chinese Academy of Medical Sciences & Peking Union Medical College, Beijing, 100730 China

**Keywords:** Human parvovirus B19, Infection, Acute respiratory distress syndrome, Cardiac surgery

## Abstract

**Background:**

Infection with human parvovirus B19 (PB19) is very common in pediatric patients. Symptoms and signs depend on the infected patient’s immune and hematopoietic status and can range from an asymptomatic condition to life-threatening disease.

**Case presentation:**

A 69-year-old man received elective mitral valvular replacement and tricuspid valvuloplasty under cardiopulmonary bypass and suffered acute respiratory distress syndrome on postoperative day 8. Through the detection of positive serum IgM and human PB19-specific nucleic acids in serum and bronchoalveolar lavage fluid via metagenomic next-generation sequencing (mNGS), acute human PB19 infection was confirmed. The patient was ventilated and the pulmonary infiltration was attenuated six days later.

**Conclusion:**

A combination of serum human PB19 DNA by mNGS and positive serum human PB19 IgM could provide higher diagnostic sensitivity for acute human PB19 infection. The method of mNGS may be a new choice for detecting rare or atypical pathogens in severe complicated pneumonia. The infection of human PB19 was possibly self-limited.

## Background

Human PB19 is a small nonenveloped single-stranded DNA virus that is the only known parvovirus that affects humans [1]. It was first discovered in 1975, and recent clinical research has found that the infection is associated with human diseases [2]. Human PB19 has been recognized to be related to childhood erythema infectiosum (fifth disease), arthropathy, and severe anemia [1, 3]. However, acute respiratory distress syndrome (ARDS) caused by human PB19 infection after cardiac surgery is rarely reported. Here, we report a patient with ARDS associated with human PB19 in a cardiac intensive care unit (ICU).

## Case presentation

A 69-year-old man with a history of mitral valvuloplasty 11 years prior and esophagectomy (esophageal cancer) 4 years prior received elective mitral valvular replacement and tricuspid valvuloplasty under cardiopulmonary bypass (CPB). The patient suffered dysfunction of the left limb after the operation, and a CT scan showed acute cerebral infarction. He was awake on postoperative day (POD) 3 and extubated on POD 4.

The patient’s temperature increased to 38.5 °C in the morning of POD 8, with hypoxemia, heart rate of 115 beats per minute, respiratory rate of 30 breaths per minute, and a blood pressure of 130/62 mmHg with norepinephrine (0.07 µg/kg/min) infusion. Pulmonary auscultation revealed bilateral basal crackles. Arterial blood gas analysis with a noninvasive V60 ventilator (PHILIPS, New Zealand) (IPAP: 16 mmHg, EPAP: 8 mmHg, FiO_2_: 100%) at that time showed pH 7.244, PaO2 84.7 mmHg, PaCO2 53.6 mmHg and PaO2:FiO_2_ (P/F) < 100 mmHg. Other laboratory data included a white blood cell count of 15.04 × 10^9^/L with 92% neutrophils and 2.1% lymphocytes, platelet count of 81 × 10^9^/L, hemoglobin of 78 g/L(preserved red cell 2U), hematocrit of 21.9%, blood lactate of 1.5 mmol/L, and blood glucose of 10.9 mmol/L. Immunologic condition evaluated on POD 11, showed decreased count of CD4 + and CD8 + lymphocytes (CD4 + : 107/µl, CD8 + : 42/µl), as well as decreased count of B lymphocytes and natural killer cells.

**Fig. 1:**
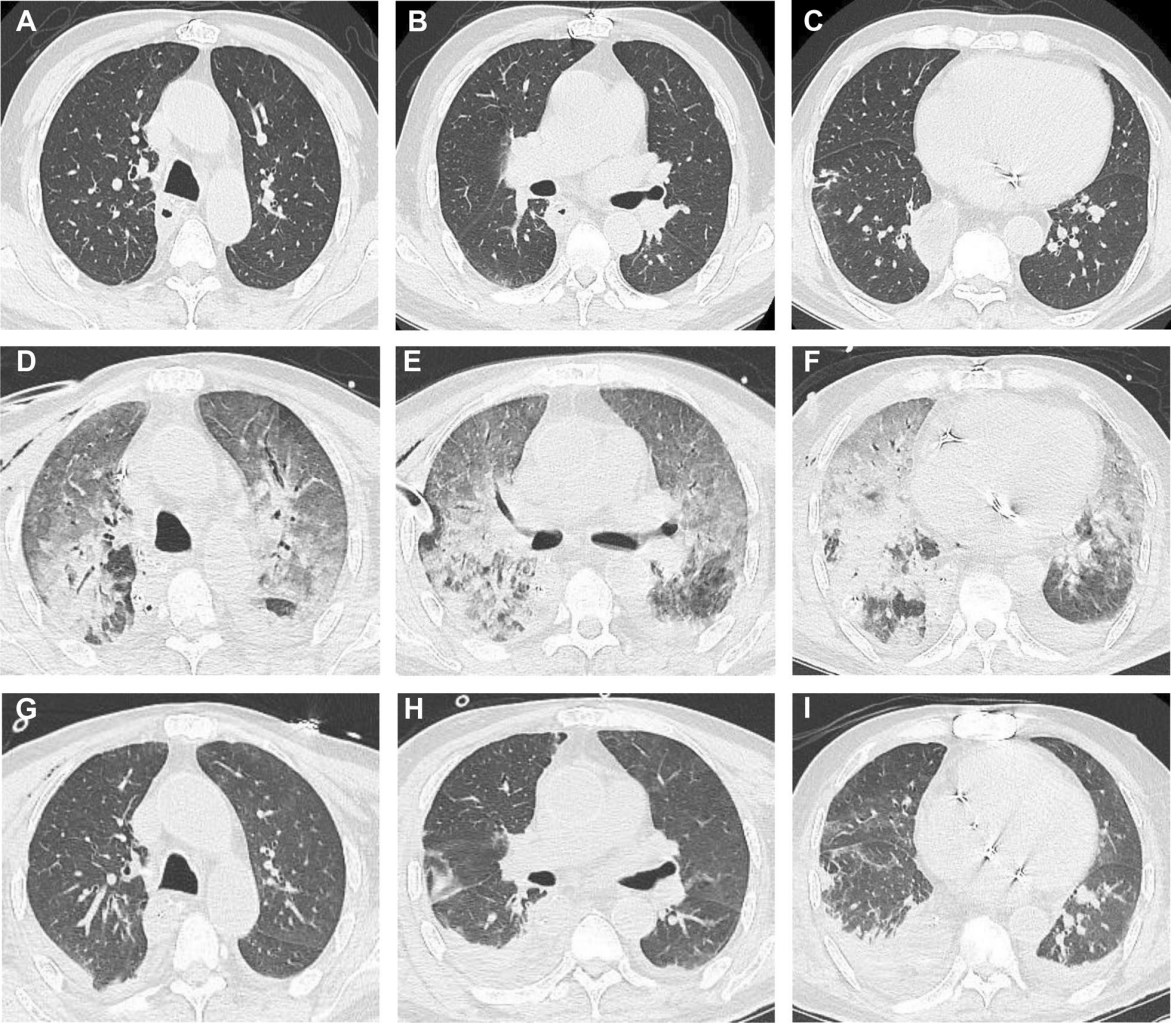
The changes of chest CT. **A**–**C** on hospital admission. **D**–**F** 8 days after operation when the patient was reintubated. **G**–**I** 14 days after operation and 6 days after reintubation

Chest radiographs showed ground glass-like changes in the lung fields. A CT scan of the chest showed diffuse bilateral alveolar infiltration and consolidation (Fig. 1D–F), compared with that on hospital admission (Fig. 1 A–C). Diagnosis of myocarditis or heart failure was not considered because transthoracic echocardiogram measurement was normal..

ARDS was confirmed by P/F and CT. Endotracheal reintubation and mechanical ventilation (840 ventilator system, NELLCOR PURITAN BENNETT, Ireland) were performed due to respiratory failure with bilevel mode (PEEP_high_: 18 mmHg, PEEP_low_: 10 mmHg, FiO_2_: 100%). Bronchoscopy was performed, which revealed a large quantity of clear serous secretions.

**Fig. 2:**
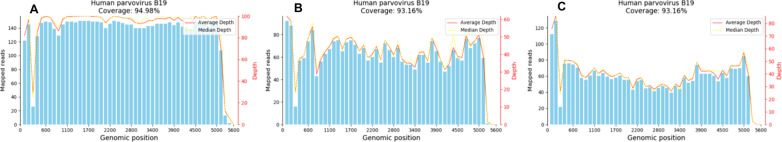
mNGS for human parvovirus B19 in the early phase and the recovery phase. **A** mNGS of the serum (8 days after operation when the patient was reintubated). **B** mNGS of the BALF (8 days after operation when the patient was reintubated). **C** mNGS of the serum (14 days after operation and 6 days after reintubation). *mNGS* metagenomic next-generation sequencing, *BALF* bronchoalveolar lavage fluid

To identify the pathogen as soon as possible, we did blood culture and sputum culture before antimicrobial administration. Then detections of mNGS in serum and bronchoalveolar lavage fluid (BALF) were performed. Human PB19-specific nucleic acids were detected in both mNGS samples (Fig. 2A, B). Confirmatory examination showed that human PB19 IgM in serum was positive, while IgG was negative (Table 1). Meanwhile, ARDS of the patient occurred during the Corona Virus Disease 2019 (COVID-19) pandemics, the new coronal nucleic acid and antibodies were all negative. So, diagnosis of COVID-19 was excluded.

**Table 1:** The changes of IgM and IgG in serum

	IgM*(index)	IgG*(index)	Normal range
Day 11^a^	3.0	< 0.10	0–0.9
Day 22^b^	17.0	8.3	0–0.9

^a^11 days after operation and 3 days after re-intubation

^b^22 days after operation when the patient was discharged

*parvovirus B19 antibody

Empiric antibiotic administration (vancomycin and meropenem) was performed after sampling of blood culture and sputum culture, and discontinued until negative blood culture and sputum culture. The patient accepted prone position ventilation and thymopentin.

The patient’s P/F began to increase with radiographic improvement after three-day ventilation. The CT manifestation of chest improved greatly (Fig. 1G–I), then the patient was extubated after six-day ventilation. The patient was discharged from the ICU on POD 15 and discharged home on POD 22. With the improvement, the mNGS in serum showed reduced sequence numbers of parvovirus DNA (Fig. 2C). Before discharge home, the human PB19 IgM and IgG in serum were both positive (Table 1). We followed up the patient at one year, he was alive and in a good condition.

## Discussion

Diseases with human PB19 infection are common in pediatric patients. It is likely that more severe illness occurs in adults than in children, and 80% of adults have comorbid arthralgias or arthritis. The virus is spread by respiratory droplets, secondary infection through household contacts, or blood product treatment, especially factor VIII and factor IX concentrates [4].

Respiratory involvement due to human PB19 has been documented [5–7] (especially in patients with immune suppression and in children). Two previously reported cases suffered dry cough and interstitial pneumonia [8]. However, ARDS caused by human PB19 appears extremely rare in adults at perioperative period. The evidence supporting acute human PB19 infection in our case was derived from the presence of human PB19-specific nucleic acids (in serum and BALF) and positive serum human PB19 IgM, and no other proven infection [9]. Furthermore, there was a significant alteration in serum IgM and IgG. In the early phase, IgM was positive, while IgG was negative. With the clearing of pulmonary infiltration, increased IgM was detected and IgG turned positive. It is usually considered that IgM is present 7–10 days following infection which indicates acute infection, while IgG is protective antibody which indicates past infection. Human PB19 DNA in serum appears 2–3 days after acute infection. But human PB19 DNA has been shown to persist in serum and synovial fluid for many years, which cannot be indicative of an acute infection. Through mNGS, a new choice for finding rare or atypical pathogens in severe complicated pneumonia [10], we narrowed the scope of suspected pathogens quickly. Thus, a combination of serum human PB19 DNA by mNGS and serum human PB19 IgM could provide higher diagnostic sensitivity for acute human PB19 infection.

This patient’s immunity level was low during his ICU stay. Although the patient had tumor history, he did not receive chemotherapy before and after cardiac surgery. It is considered that the immune dysfunction has relationship with cardiac surgery injury, CPB, blood loss and malnutrition [11]. Finally, immunodepression may increase susceptibility to infection [12]. We should be vigilant against virus infection.

How the reported patient became infected is unclear. During the perioperative period, another patient (suspected patient) in the same surgical ward had the same manifestations. The severe pulmonary infiltration of this suspected patient was accentuated in one week, and human PB19 was detected in the serum by mNGS after discharge home. Both of the patients had blood transfusions, but the two patients had different blood types. It was impossible that they had the same lot of blood transfusion.

Outbreaks of human PB19 infection among patients have been reported in adult surgical wards [13]. Although there was no evidence revealing the relationship between these two patients, no more cases appeared after terminal disinfection of the ward. These two patients from different areas were followed up for one month, there was no outbreak in their communities.

The precise mechanism of human PB19 pneumonia is unclear. It is uncertain whether lung injury is caused by direct infection or a secondary immune response from the host. The infection is usually self-limited. Specific antiviral therapy is unavailable to treat human PB19 infection [1]. Intravenous immunoglobulin (IVIG) has been proven to be effective in pure red cell aplasia or immunosuppression [14]. However, the efficiency of IVIG in immunocompetent patients is unclear [3]. Support therapies such as intubation, prone positioning, and protective ventilation were important in this case.

In conclusion, human PB19 should be considered in the differential diagnosis of unknown pneumonia for immunodeficiency patients. The determination of human PB19 antibodies, especially positive serum IgM, and the human B19-specific nucleic acids in serum should be examined for the accurate diagnosis of acute human PB19 infection [9, 15]. mNGS may be a new choice for finding rare or atypical pathogens in severe complicated pneumonia, especially for some virus [10].

## Data Availability

The datasets used and/or analyzed during the current study are available from the corresponding author on reasonable request.

## Abbreviations

PB19: Parvovirus B19
mNGS: Metagenomic next-generation sequencing
ARDS: Acute respiratory distress syndrome
ICU: Intensive care unit
POD: Postoperative day
CPB: Cardiopulmonary bypass
BALF: Bronchoalveolar lavage fluid
COVID-19: Corona Virus Disease 2019
IVIG: Intravenous immunoglobulin
